# 

**Published:** 1909-12-25

**Authors:** 


### Gifts and Bequests.

Mr. William McKewan has left in his will £100 to the Bromley Cottage Hospital.

Miss Charlotte Mary Phillips has bequeathed £100 to the Norwood Cottage Hospital.

Mrs. Hannah Matthews, who died on November 6, has left £100 each to the Royal United Hospital, Bath, and the Mineral Water Hospital, Bath.

Mr. John Thomas Slater has left £1,050 each to the Great Northern Central Hospital for the endowment of a "Slater" bed, and to the City of London Hospital for Diseases of the Chest for a like purpose; £1,000 to the Hornsey Cottage Hospital for a "Slater" bed.

Dr. Charles Graham, of Hastings, at one time Professor of Chemistry at University College, London, subject to various bequests, has left the residue of his estate, probably about £35,000, to the Senate of the University of London, to found a fund to be known as the "Charles Graham Medical Research Fund," in aid of any research carried on by a teacher or student of the School of Advanced Medical Studies of the University College Hospital for the prevention, cure, or alleviation of human disease or suffering; provided that, shall any student or teacher conduct any research which the committee consider of sufficient merit for the prevention, cure, or alleviation of human disease and suffering, they shall award to such one a gold medal of appropriate value. The committee is also to pay to such teacher or student of the said school conducting such research a sum not exceeding £200 per annum for two years, such person to be known as the "Charles Graham Student in Pathology."

The will of Miss Adelaide Annie Bankes contains bequests of £1,000 to the Wimborne Cottage Hospital, and £500 to the Swanage Cottage Hospital.

Miss Laura Batty has left £100 to the Hospital for Incurables at Putney; £50 each to the Mount Vernon Hospital for Consumption and Diseases of the Chest, and to University College Hospital.

The trustees of the Annie Zunz Fund have given a further donation to the West Ham and East London hospital of £1,000 to furnish the ward, provided by that fund at a cost of £3,000, in the new extension.

The late Sir John Baker, M.P. for Portsmouth, has left £500 to the Portsmouth, Portsea, and Gosport Hospital for the Convalescent Home in connection therewith; and £100 to the Portsmouth Eye and Ear Infirmary.

The Corporation have made recently the following grants:—Royal National Orthopædic Hospital, £100. The St. John's Hospital for Diseases of the Skin has received £500 for the naming of a ward to be called the "Annie Zunz" ward. The Chelsea Hospital for Women has received £10 10s. from the Broderers' Company.

The late Sir Benjamin L. Cohen has left £200 to the Jews' Hospital and Orphan Asylum; £300 to St. Bartholomew's Hospital, and £100 each St. Mary's Hospital, Paddington; the Hospital for Consumption, Brompton; the London Homœopathic Hospital; the Royal Hospital for Incurables, Putney; the Mount Vernon (Hampstead) Hospital for Consumption; the Great Northern Central Hospital, Holloway Road; the East London Hospital for Children and Dispensary for Women, Shadwell.

### THE QUESTION BOX.

SPECIAL NOTICE.—Questions of interest affecting the honorary medical staff and hospital administration in all departments are invited. When any point arises we hope our readers will formulate it in a question and so co-operate to make this column of real value and interest. In this way the experience of the best-managed hospitals should be made helpful to the whole hospital world. We shall welcome the contribution of both questions and answers.

N.B.—The publication of an answer in this column does not necessarily preclude the publication of subsequent answers to the same question, for a question may involve points which require to be threshed out and fully discussed. Answers, if published, will be paid for at scale rates.

### THE COMMITTEE OF MANAGEMENT AND THE HONORARY MEDICAL STAFF.

### THE QUESTION OF REPRESENTATION.

QUESTION 1: Is it desirable that the Honorary Medical Staff should have seats on the General Committee, or that they should meet as a Medical Board and recommend to the General Committee any change they may consider necessary?

### A London View.

By Mr. CONRAD W. THIES, Secretary, Royal Free Hospital.

It is desirable that the honorary, or visiting, medical staff should be represented on the General Committee; also that they should meet independently as a medical board and make their recommendations to the General Committee. The question of the representation of the honorary medical staff upon the governing and executive committee of hospitals has been the subject of considerable discussion in recent years, and one well-known instance, where the medical staff of a London voluntary hospital successfully struggled for a voice in the management, will occur to most readers. Under existing arrangements the visiting medical staff, with very few exceptions, do not receive any direct remuneration for the services which they render to the voluntary hospitals, although it is generally recognised that they derive certain substantial indirect advantages from being appointed to the staff of these institutions, otherwise these appointments would not be so keenly sought after. The admission of the patients is almost entirely in their hands, and they have it in their power very materially to affect the expenditure. It is therefore very desirable that they should be afforded the opportunity of becoming practically conversant with administrative details, of which many medical men have no experience. The various subjects which come up for consideration by the executive are frequently of a character whereon the lay members will be greatly assisted by the advice of a member of the medical staff, who is practically conversant with the work, and is therefore able to point out the bearing of any particular question as it may affect the general working of the institution. A lay executive is liable at times to arrive at decisions on what appear to be minor administration details without realising how such decisions will affect the general well-being of the institution, whereas the opinion of a member of the medical staff will often prevent such an erroneous judgment. These considerations have convinced me that it is desirable that the medical staff should be represented upon the executive, while on the other hand it is most undesirable that they should have a preponderating influence thereon.

### A Leicester View.

By Mr. HARRY JOHNSON, House Governor and Secretary, Leicester Infirmary.

The board of governors have found it very desirable that the honorary medical staff should have seats on the board, and great gain has resulted from the honorary medical staff meeting as a medical committee—*e.g.* to recommend candidates for house appointments, to consider any changes which it may be considered desirable to introduce into the administration of the institution.

### A Scottish View.

By a MEDICAL SUPERINTENDENT.

At the last representative meeting of the British Medical Association held in Belfast, the following resolution was unanimously passed:—

"That, in the opinion of this representative meeting, the association of officially recognised representatives of the local medical profession in the promotion and general management of hospitals and other medical charities is desirable in the interests of those institutions, as well as of the profession."

In addition to this representation from the medical profession, it is well that the staff should be constituted into an Advisory Committee in order that the general board of the hospital may have the benefit of their opinion on matters which may present themselves from time to time. By this arrangement every member of the medical staff has an equal opportunity of expressing his opinion, as it would be inconvenient, if not impossible, for every member of the medical staff to give regular attendance to all the board and committee meetings.

### COAL DELIVERIES AND THEIR WEIGHT.

QUESTION 1: What percentage of reduction in the coal bill of an institution may be expected from weighing coal on delivery before accepting it?

ANSWER: Nearly 10 per cent.

By a MEDICAL SUPERINTENDENT OF AN INSTITUTION.

For obvious reasons I prefer to remain anonymous; but I can vouch for the following figures: Annual coal consumption before weighing on delivery, roughly 8,000 tons; annual saving after each cart load was weighed on a small weighbridge (cost about £10)—a note of the weight being made on delivery note by the engineer on duty—700 tons. With the Editor's permission, I should be interested to hear the experience of others in this regard.

*** Further experiences on this subject are invited.—ED. *T.H.*

### WOMEN ON HOSPITAL BOARDS.

QUESTION: Is it desirable that women should be appointed as members of the Governing and Executive Committees of voluntary hospitals?

AT the fifty-fourth annual festival of the Poplar Hospital for Accidents Sir Hudson Kearley, M.P., said that its income had increased from £4,000 a year to £14,000 a year, and its reserve fund from £8,000 to £50,000, and 50,000 cases were treated annually as compared with 20,000 two years ago. The Secretary announced that the total contributions amounted to £2,769, including £105 from the Port of London Authority.

Dr. C. Hubert Roberts, M.D., F.R.C.S., has been appointed a physician to in-patients; Dr. R. D. Maxwell, M.D., F.R.C.S., has been appointed a physician to out-patients; Mr. Thomas Crisp English, M.B., F.R.C.S., has been appointed surgeon (non-obstetric), all at Queen Charlotte's Lying-in Hospital.

THE HOSPITAL December 25, 1909.

*Name* ..................................

*Address* ..................................

..................................

This Coupon must accompany manuscript or contributions intended for The Hospital.

